# Isotretinoin-Induced Raynaud's Phenomenon

**DOI:** 10.7759/cureus.97699

**Published:** 2025-11-24

**Authors:** Alya Al Azri

**Affiliations:** 1 Department of Dermatology and Venereology, Sinaw Hospital, Ministry of Health, Al Mudhaibi, OMN

**Keywords:** acne, case report, drug-induced raynaud's phenomenon, isotretinoin, raynaud's phenomenon, vascular symptoms

## Abstract

Raynaud's phenomenon (RP) is characterized by color changes in the fingers and toes, typically triggered by cold or stress, and can occur as either a primary or secondary condition. Secondary Raynaud's phenomenon is associated with various underlying diseases, medications, and environmental factors. Isotretinoin, a widely used treatment for acne, is known for its potential side effects, but drug-induced RP remains an underrecognized complication. We present the case of a 20-year-old female who developed RP following the initiation of isotretinoin therapy for moderate acne, a reaction described as dose-dependent in some patients. This case highlights the potential for isotretinoin to induce RP and the importance of dose adjustment or discontinuation in managing this side effect.

## Introduction

Raynaud’s phenomenon (RP) is a vasospastic disorder characterized by episodic ischemia of the extremities, typically triggered by cold or emotional stress. RP can be classified as primary (idiopathic) or secondary, often associated with underlying conditions such as autoimmune diseases or drug exposure [1].

Medications such as beta-blockers, chemotherapeutic agents, vasoconstrictive drugs, and serotonergic agents have been implicated in the onset of RP [2]. Isotretinoin, a retinoid authorized by the FDA, is a drug treating acne vulgaris; however, it is known to cause several adverse effects, restricting its use [3]. Recent pharmacovigilance reports have suggested a potential link between isotretinoin and RP [4].

## Case presentation

**Figure 1:**
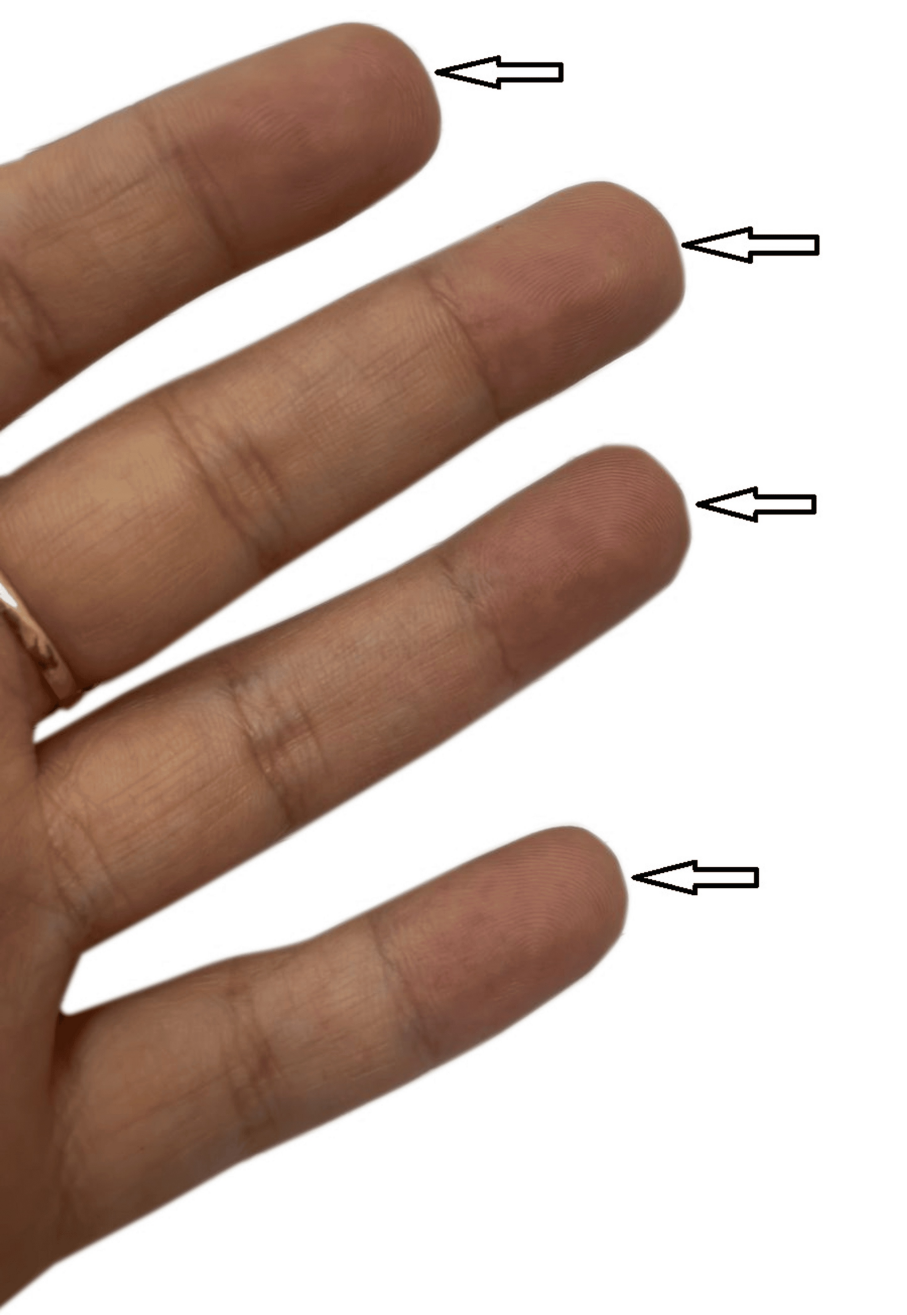
Faint bluish discoloration on the distal third of most fingers.

**Figure 2:**
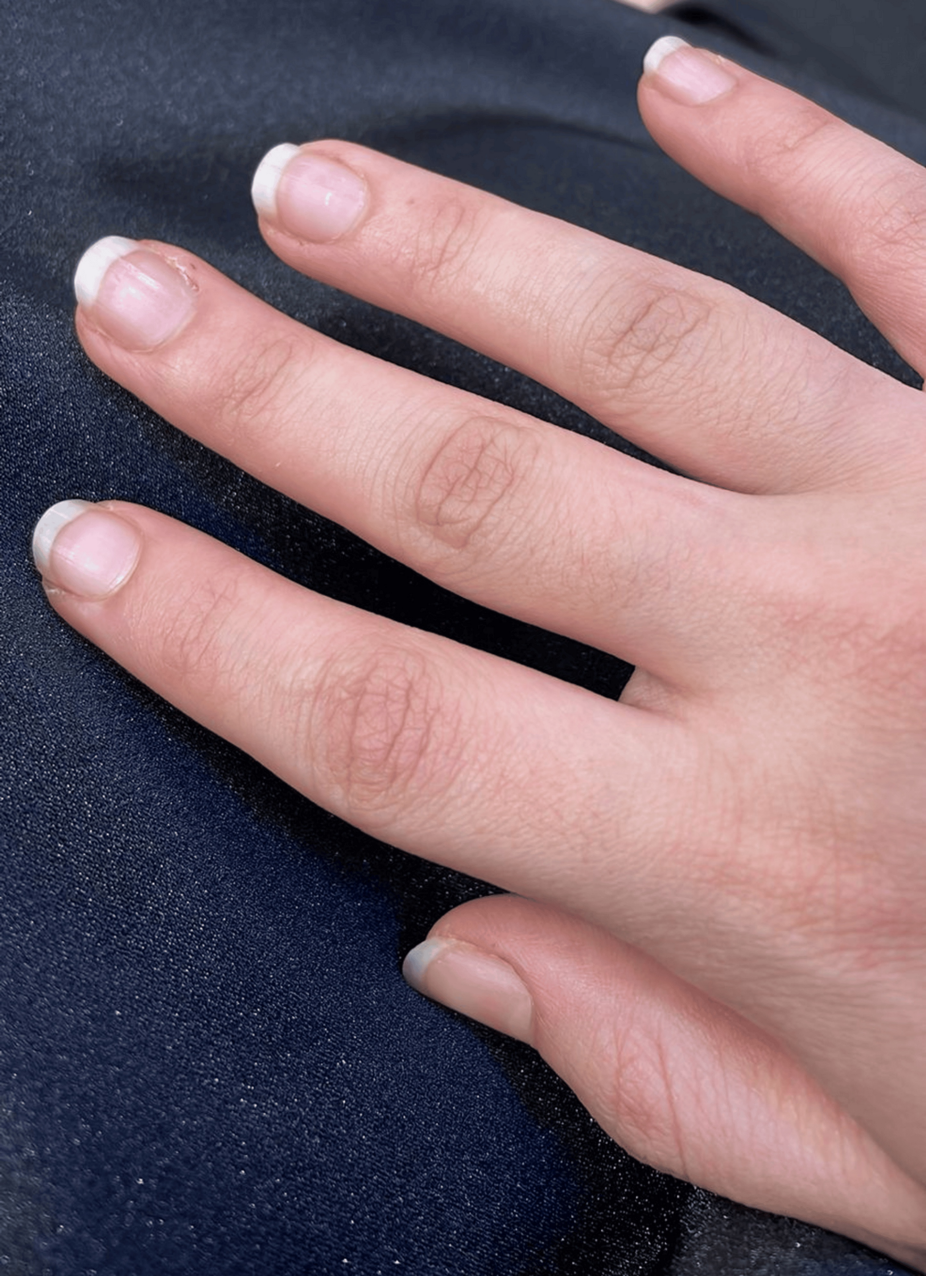
A bluish discoloration of the nail folds.

**Figure 3:**
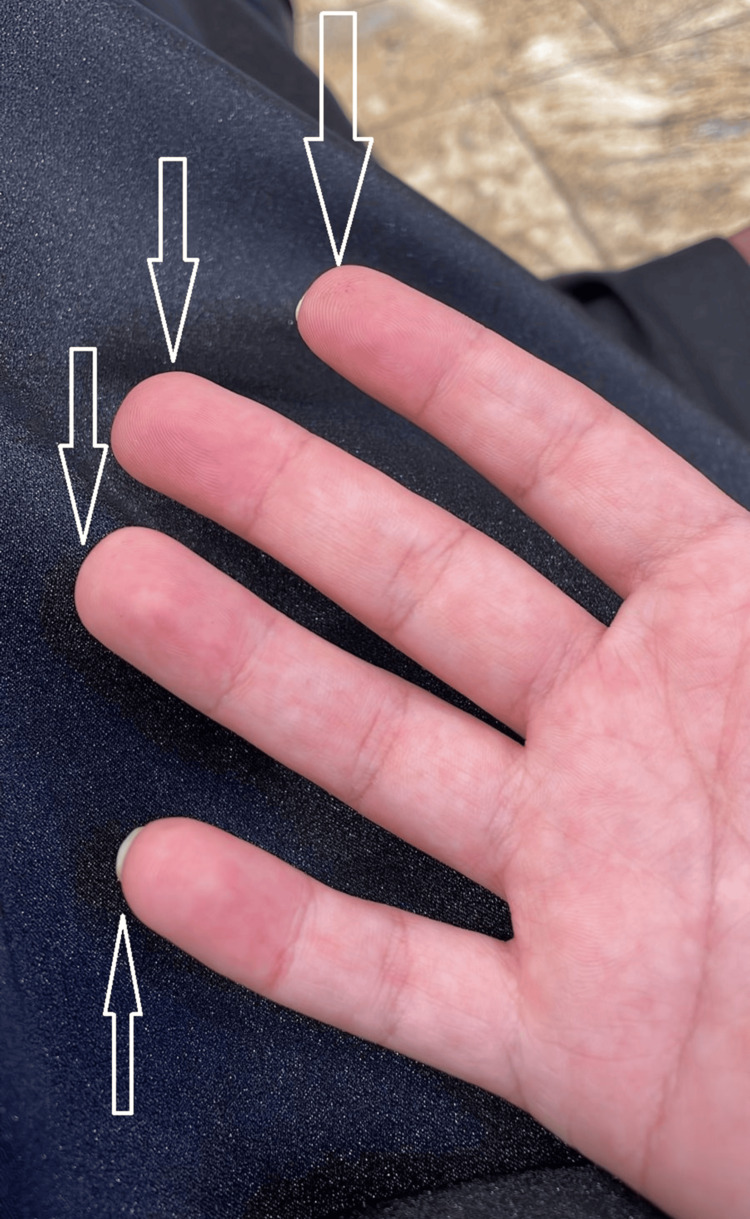
Erythema on the palmar aspect of the distal fingers of the left hand.

A 20-year-old female with moderate acne started isotretinoin therapy at 30 mg daily (0.5 mg/kg) for two weeks, then the dose was increased to 40 mg (0.6 mg/kg) for two weeks. She developed intermittent pale-red-bluish discoloration in her fingers, along with a sensation of coldness and discomfort with tingling and numbness. She had no history of vascular issues. Isotretinoin was stopped for a week, and symptoms resolved completely; however, the patient insisted on continuing treatment. Due to institutional limitations, autoimmune serology was not performed. Isotretinoin was restarted at a dose of 20 mg (0.3 mg/kg), and the patient was fine without complaint for three weeks. She then increased the dose herself to 30 mg (0.5 mg/kg) for a week. The same vascular symptoms recurred, so the dose was tapered to 20 mg (0.3 mg/kg) without complaint. The patient showed no recurrence of RP while she was on a 20 mg (0.3 mg/kg) dose, which continued for seven months. On clinical examination, she had faint blue discoloration of the distal third of most fingers (Figure 1). A bluish discoloration of the nail folds was also noted (Figure 2). The patient provided a photograph showing an episode of hyperemia affecting the distal part of all fingers (Figure 3). Based on the history and clinical examination, the diagnosis of RP was made. A causality assessment using the Naranjo Adverse Drug Reaction Probability Scale revealed a definite link (score 10) between isotretinoin and RP.

## Discussion

RP is a condition characterized by a temporary vasospasm of arteries and arterioles, leading to changes in skin color in the affected extremities. It typically follows a three-phase pattern: pallor, cyanosis, and redness. It can be associated with numbness, paresthesia, and pain in the fingers.

There are two types of RP: primary and secondary. Primary RP occurs without an underlying cause, while secondary RP is linked to other medical conditions such as connective tissue diseases, vasculitis, endocrine disorders, or exposure to certain medications [5,6].

Among the various causes of secondary RP, drug-induced RP (DI-RP) is an underexplored category due to the lack of large-scale epidemiological studies, underreporting of cases, and the complex interplay between drug pharmacodynamics and individual susceptibility. Many cases of DI-RP may be misattributed to underlying conditions or environmental factors, leading to gaps in clinical awareness and research focus [2].

The pathogenesis of RP is not fully understood. However, multiple factors have been proposed, including vascular dysregulation, endothelial dysfunction, and an imbalance in vasodilatory and vasoconstrictive substances (e.g., prostaglandins and nitric oxide). Some individuals may also have a genetic predisposition [2,7-9].

In addition, neurovascular contributions such as overactivation of the sympathetic nervous system lead to excessive vasoconstriction [10].

Several drug classes have been implicated in the development of RP, with prevalence rates varying among drug types [2].

Isotretinoin is a retinoid primarily used for severe acne. It exerts its effects by modulating gene expression involved in cell differentiation, proliferation, and apoptosis [4]. However, isotretinoin has been associated with vascular changes, including endothelial dysfunction by inhibiting nitric oxide production, leading to impaired vasodilation [7]. Several studies have demonstrated that retinoids influence vascular smooth muscle cell function and may interfere with angiogenesis [11,12]. Case reports suggest an increased risk of thrombotic events and vasculitis associated with isotretinoin use [13-15]. Several pieces of evidence have indicated a potential link between retinoids and the coagulation system [12].

Given the emerging evidence of retinoid-induced vascular side effects, including RP, clinicians should monitor patients using retinoid therapy for evidence of such complications and follow established guidelines for managing DI-RP.

## Conclusions

DI-RP remains an underrecognized but significant clinical condition. Isotretinoin, traditionally known for its dermatologic applications, has been implicated in vascular complications, including RP. While direct causation is yet to be firmly established, pharmacovigilance data and case reports suggest a strong association. Future research should focus on elucidating the precise mechanisms linking isotretinoin to vascular dysfunction, including potential genetic predispositions, long-term follow-up studies to assess chronic vascular effects, and the development of targeted interventions to mitigate associated risks. Additionally, large-scale epidemiological studies could provide more definitive insights into the prevalence and severity of isotretinoin-induced RP.
